# An invisible workforce: the neglected role of cleaners in patient safety on maternity units

**DOI:** 10.1080/16549716.2018.1480085

**Published:** 2019-01-15

**Authors:** Suzanne Cross, Giorgia Gon, Emma Morrison, Koasar Afsana, Said M. Ali, Tina Manjang, Lamin Manneh, Atiya Rahman, Deepak Saxena, Kranti Vora, Wendy J. Graham

**Affiliations:** a The Soapbox Collaborative, Aberdeen, UK; b Department of Infectious Disease Epidemiology, London School of Hygiene and Tropical Medicine, London, UK; c Health, Nutrition & Population Programme, BRAC, Dhaka Division, Dhaka, Bangladesh; d Pemba Public Health Laboratory Ivo de Carneri, Zanzibar; e Horizons Trust Gambia, Fajara, The Gambia; f Ministry of Health & Social Welfare, Banjul, The Gambia; g Research & Evaluation Division, BRAC, Dhaka Division, Dhaka, Bangladesh; h Indian Institute of Public Health, Ahmedabad, Gujarat, India

**Keywords:** Infection prevention, cleaners, healthcare associated infections, low and middle income countries, environmental hygiene

## Abstract

Hospital cleaning has been shown to impact on rates of healthcare-associated infections (HCAIs) and good environmental hygiene is critical to quality care, yet those tasked with the role of ensuring a safe and clean environment often go unrecognised as members of the healthcare workforce. Sepsis is a leading cause of maternal and newborn death, a significant proportion of these cases are estimated to be due to HCAIs. Deliveries in health institutions have now reached 75% globally, and in low and middle income countries the corresponding increased pressure on facilities  has impacted both quality of care provided and quality of the birth environment in terms of infection prevention and control (IPC) and HCAIs. The paper discusses the neglected role of health facility cleaners, providing evidence from the literature and from needs assessments conducted by The Soapbox Collaborative and partners in Bangladesh, India, The Gambia and Zanzibar. While not the primary focus of the assessments, common themes emerged consistently pointing to institutional neglect of cleaning and cleaners. The paper argues that low status within facilities, wider societal marginalisation, lack of training, and poor pay and working conditions contribute to the lack of prioritisation placed on health facility environmental hygiene. With increased international attention focused towards health facility water, sanitation and hygiene and a growing focus on IPC, now is the time to address the neglect of this frontline healthcare workforce. We propose that provision of and improved training can enable the recognition of the valuable role cleaning staff play, as well as equipping these staff with the tools required to perform their job to the highest standard. In addition to training, wider systems changes are necessary to establish improvements in environmental hygiene and the role of cleaning staff, including addressing resource availability, supportive supervision, and an increased emphasis on preventative healthcare.

## Background

The 2015 Millennium Development Goals’ target of 90% of deliveries taking place  with skilled attendants was not achieved. Improvements in coverage were nevertheless made with three quarters of deliveries now occurring in health facilities [1]. Consequently however, in low and middle income countries (LMICs), exacerbation of staff shortages, increased work pressure, and poor infrastructure and supplies have impacted quality of both delivery care and the birth environment [2]. As a result, efforts to reach Sustainable Development Goal targets of further reductions in maternal and newborn mortality are being hampered. Sepsis is a leading cause of maternal and newborn death, making up between 4 and 56% of all causes of death among hospital born babies [3]. The contribution to these deaths from healthcare associated infections (HCAIs) is estimated to be significant [4]. Of these sepsis deaths 75% occurring in South East Asia and Sub-Saharan Africa where exposure to unhygienic practices and environments exacerbate figures [3]. The increased risk to mothers and newborns of iatrogenic infections are well known [5]. Despite this, primary prevention of infections through water, sanitation and hygiene (WASH) and infection prevention and control (IPC) is often lacking [6].

**Figure 1.**
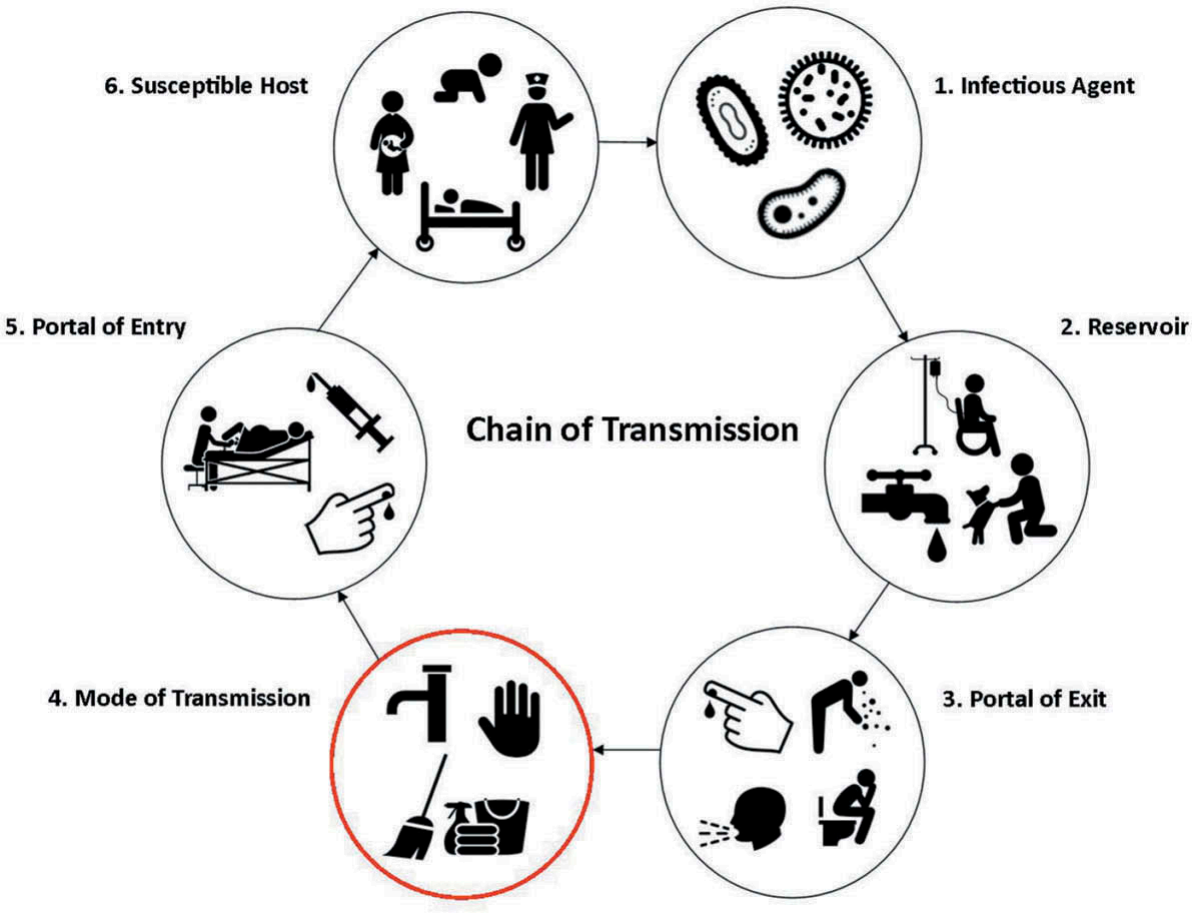
Chain of transmission.

The contribution of the physical environment in IPC must be considered as both a direct infection risk to mothers and newborns and an indirect infection risk via contamination of clean hands [4,7–9] (Figure 1). Studies in LMICs show that environmental surfaces (hand wash basins, mattresses, etc.) have contributed to outbreaks in neonatal units with gross contamination of surfaces a potential source of infection [3,10]. Swab sampling of high-risk sites in delivery rooms in Bangladesh found *Staphylococcus aureus* commonly found on the delivery room door handles and maternity ward beds [11]. Similarly in Zanzibar, Tanzania maternity beds of surveyed facilities were found to be highly contaminated, with multiple organisms also found on mops and cleaning cloths [12]. This poor state of hygiene has also  been suggested to contribute to the overuse of antibiotics in LMICs [6].

For a safe environment to be established and maintained, the focus must include those personnel primarily tasked with cleaning [13], referred to by a variety of context-dependent terms such as orderlies and domestic assistants. For the purposes of this paper we use the term ‘cleaners’ or ‘cleaning staff’.

Although an understudied area, evidence demonstrates the impact of improved hospital cleaning on infection rates [14]. Despite their key role in IPC however, little reference is made to cleaning staff in either the literature or IPC/environmental hygiene guidelines. A clear example of this omission comes from the absence of cleaners among key stakeholders listed in the WHO Essential Environmental Health Standards in Health Care, often referred to as the gold standard [15]. What literature does exist points to a generalised neglect of cleaners and a lack of recognition; cleaners have little control over their role, responsibilities and work environment [16]. Studies of other cadres have found these issues to affect workers’ job satisfaction, performance and health [17].

This neglect is perpetuated due to the low societal value attached to cleaning, frequently seen as menial, dirty work. Cleaning is often reserved for the marginalised; individuals performing cleaning roles are often linked to disadvantaged socio-economic groups [18]. This becomes more complex in cultures where birth is seen as ‘polluting’ [19]. Thus, marginalised individuals are performing a marginalised role.

## Soapbox Collaborative Needs Assessments

To illustrate the arguments made above and provide further evidence of the marginalisation of cleaning staff, examples are provided from needs assessments conducted by The Soapbox Collaborative, an evidence-based charitable trust committed to ensuring mothers and newborns avoid HCAIs at the time of delivery. Soapbox, with partners, has conducted assessments of WASH and IPC (including environmental hygiene) in maternity units in a number of diverse LMICs – India, Bangladesh, Zanzibar and The Gambia.

The assessments took a mixed methods approach to capture evidence of the status and drivers of WASH and IPC on maternity units. In each country a questionnaire was administered to the head nurse or equivalent of the maternity unit gathering information on the healthcare organisation and operations, (human) resources, and IPC and healthcare practices. Semi-structured interviews were conducted with a total of 105 stakeholders including management, healthcare professionals and cleaning staff across the four countries, and a combined total of 56 facilities surveyed – community health centres, primary health centres, clinics, district, and sub-district hospitals and included both private and public facilities, all conducting deliveries. Further details, including a description of the methodology and published findings for the Zanzibar, India and Bangladesh studies, are described in [11,12], methods for data collection and analysis in The Gambia followed a similar format.

While the purpose of the needs assessments was not to compare across countries, a number of common themes relating to cleaning staff were identified, including around training and cleaners’ status within the workplace, as summarised below.

Across the four countries at least half of the facilities did not provide any form of IPC training for non-medical staff, including cleaners (Table 1). Of those facilities providing training, the interviews revealed that training was not comprehensive; reaching only a small number of cleaners and was generally limited to training in hand washing and surface cleaning.

**Table 1.** Availability of IPC training for cleaning staff across countries

		IPC training available for cleaning staff		
Country	No. of facilities	Yes	No	Interviewee did not know
Bangladesh	8	4 (50%)	4 (50%)	-
India	7	-	7 (100%)	-
The Gambia*	4	-	4 (100%	-
Zanzibar	37	13 (35%)	21 (57%)	3 (8%)

*included labourers, laundresses and maintenance staff

Interview findings across countries revealed that training was deemed unnecessary by some, including cleaners themselves. They indicated approaching cleaning of the maternity unit as they did their homes, not aware of the additional precautions required to ensure a ‘safe’ standard of hygiene and IPC.

‘What we do for facility cleaning is like my household cleaning… there is nothing needs to be learned by training’

Cleaner, Bangladesh

In general however, interviewees did recognise the implications of the lack of training and labelled it a significant concern:

‘Orderlies [cleaners] are a critical mass that need to be trained … they also do works that are related to IPC. If you had an orderly, and they are not trained still, there’s going to be a gap. If that gap is not bridged, it may cause a serious problem in the long run…’

IPC Focal Point, Ministry of Health, The Gambia

‘Yes, cleaners should be taken as part of the health work force… Some don’t know how to protect themselves but if trained they will know how to protect themselves…’

Healthcare Professional, Zanzibar

Cleaners were often burdened with tasks unrelated to their primary role, making up for a lack of skilled staff. This is of particular concern considering findings from Bangladesh and Zanzibar which reported the involvement of untrained cleaning staff in patient care.

*‘We are allocated shifts on our own with no any nurse present. I deliver women, I give injections, I prescribe medicines, we also examine pregnant women. I have not received any training*.’

Cleaner, Zanzibar

In India, according to managers of both public and non-public facilities, cleaners should be ‘strengthened’ through training and paid more to ensure a high standard of work.

‘For improvement it is necessary to increase the strength of the class 4 servants [cleaners]… nobody can clean except them so the most important is to increase salary of class 4…’

Healthcare Professional, India

Low levels of pay, and dissatisfaction with this, was repeated throughout the interviews as a marker of wider marginalisation. This marginalisation was further illustrated by Oneaya (healthcare assistant) in Bangladesh who, in an interaction with one of the research team, commented on being too ashamed to introduce her mother who worked as a cleaner in the same facility. Reflecting on their participation in the assessments, another cleaner stated pointedly:

*‘Nobody wanted to know what we do and how pressurised with work we are… our work is undervalued both institutionally and socially’*.

Cleaner, Bangladesh

Disparities between cleaning staff and other members of the healthcare workforce were discussed in the interviews in India and Bangladesh where cleaners’ lack of benefits, and for some cleaning staff lack of contractual security, was reported. The need for ‘incentives’ e.g. bonuses to act as a motivating factor for cleaning staff was noted;

“For motivation some incentives should be given… then people will be motivated to do more work. They will take interest…”

Healthcare Provider, India

Interestingly however, while results from Zanzibar pointed to poor pay of cleaning staff, conditions in terms of holidays and sick leave were comparable to other cadres within the health facilities.

## Discussion

For sustained reductions in HCAIs in maternity units it is imperative that the neglect of cleaners, and thus cleaning, is addressed. However, the dearth of published evidence on the current status of environmental hygiene and the barriers faced by cleaners within LMICs is indicative of wider neglect. What evidence does exist supports the findings presented here, selected for illustrative purposes from wider work looking at WASH and IPC on maternity units.

The cleaning industry is ‘notorious’ for its poor pay and conditions [18,20]. Hospital cleaners are often devalued, at the bottom of the hierarchy and have low status within facilities [21,22]. Much of the stigmatisation surrounding cleaning and cleaning staff comes from the view of cleaning as ‘women’s work’, of little productive value [21]. With regard to childbirth, in some contexts culturally and historically women have been seen as ‘unclean’ or ‘polluted’ at the time of delivery, and believed to be dirty and weak in the postnatal period [23]. These beliefs have exacerbated negative views associated with cleaning and further marginalised those tasked with this role, particularly on maternity units. As noted by Van Hollen, ‘cultural notions of “pollution” are thought to be major threats to women’s health during delivery and the postpartum period and serve as obstacles to “modern” attitudes towards sepsis and sanitation’ [24].

These views and the poor status of cleaning staff are not limited to health facilities, however. There is a societal undervaluing of the roles and the rights of these individuals within the wider context. Small steps can nevertheless be taken to begin to tackle these issues, starting with the work environment. Productive, mutually respectful relationships between cleaning staff, healthcare providers, and the wider hospital personnel need to be fostered, and cleaners equipped with the knowledge, skills and understanding required to perform their job effectively and efficiently. Training of cleaning staff is highly relevant to the prevention of HCAIs [25] and has been linked to motivation and performance [26]. Training also has the potential to impact relationships with healthcare providers and foster recognition of cleaning staff as valued members of the workforce, as well as support cleaning staff themselves to recognise the importance of their own role in infection prevention.

Training is advocated as a key starting point, however there is a need to acknowledge that without wider systems change, combined with ongoing supportive supervision, its benefits may not be optimized (e.g. [27]). As suggested in the WHO Core Components for IPC Programmes, a good programme needs to work throughout the system and involve organisational and cultural change [27]. This needs to include an increased emphasis on preventative healthcare; the current lack of focus on which may help to explain the lack of investment in cleaners/cleaning.

While availability of resources (including cleaning materials) and staffing, among other factors, impacts on the ability to maintain a clean and safe environment, the basics of training, availability of policies and protocols and fair working conditions should be in place regardless and link inextricably to quality improvement.

On average, less than a third of facilities surveyed across India, Bangladesh, The Gambia and Zanzibar delivered formal training to their cleaning staff and interviewees spoke of the poor status of cleaners within facilities. Despite differences in numbers of facilities included in each country study, there were clear patterns with common findings across diverse contexts pointing to the likely applicability of the findings to other low-resource settings. As is suggested in the literature, limited training and the poor status of cleaning staff is not confined to the maternity unit but relates to the wider hospital setting [22]. Further research is needed to address this area more broadly with larger numbers of facilities, more nuanced analyses within countries, and differentiation between public and non-public facilities.

The state of the environment is intrinsically linked to WASH and IPC within facilities and here we have presented evidence of an institutional neglect of cleaning staff, and thus a corresponding neglect of environmental hygiene. However, only in the last few years has WASH in healthcare facilities garnered significant attention, marked by the publication of the 2015 WHO/UNICEF report of the same name and the recent Call to Action on IPC [28,29]. Combined with the push to reduce maternal and newborn death, reduce HCAIs, and address antimicrobial-resistance, there has never been a more opportune moment to turn our attention to frontline environmental hygiene and IPC workers. There is a clear need to empower these forgotten members of the healthcare workforce through raising awareness among key stakeholders, strengthening cleaners’ knowledge and practice, and tackling the institutional bottlenecks which often neglect the poor state of hygiene and the infection-related consequences, and thus this cadre. Through this, we will move closer to the ultimate goal of cleaner, safer, respectful care for mothers and newborns and a reduction in HCAIs, and provide cleaning staff with the respect, time and resources they deserve as key members of the healthcare workforce.

## Paper context

Marked increases in institutional deliveries across low- and middle- income countries over the last 5–10 years have resulted in significant pressure on facilities, impacting quality of care and infection prevention and control. Hospital cleaning contributes to the prevention of healthcare-associated infections, yet cleaners are often a marginalised cadre, with poor working conditions and recognition. A missed opportunity for improvement of environmental hygiene, as a key component of quality care, lies in cleaners’ training and empowerment.
